# Behavioural manipulation of the cabbage stem flea beetle ( *Psylliodes chrysocephala* L): Prospects for push–pull strategies in oilseed rape

**DOI:** 10.1002/ps.71010

**Published:** 2026-06-20

**Authors:** Suleiman Mustapha, Patricia A Ortega‐Ramos, Jozsef Vuts, Tariq M Butt, E. Joel Loveridge, Daniel Rüde, Samantha M Cook

**Affiliations:** ^1^ Agroecosystem Health and Pest Management Rothamsted Research Harpenden UK; ^2^ Department of Biological Sciences Swansea University Wales UK; ^3^ Translating Biotic Interactions Rothamsted Research Harpenden UK; ^4^ Department of Chemistry Swansea University Swansea UK; ^5^ Agricultural Entomology, Department of Crop Sciences University of Göttingen Göttingen Germany

**Keywords:** *Brassica napus*, companion plant, host–plant interactions, integrated pest management, plant volatile organic compounds, rapeseed, semiochemicals, trap crop

## Abstract

The cabbage stem flea beetle (CSFB, *Psylliodes chrysocephala* L) is a major pest of oilseed rape (OSR, *Brassica napus* L), especially in temperate regions where it is challenging the sustainable production of the crop. Conventional management has relied almost exclusively on synthetic insecticides, yet increasing regulatory restrictions, pest resistance and environmental concerns require the urgent development of alternative strategies. Semiochemicals, chemical cues that mediate inter‐ and intraspecific behavioural interactions, offer considerable potential for integration into push–pull pest management programmes, where combinations of attractant and deterrent or repellent stimuli are used synergistically to reduce crop pest infestation. This review synthesises knowledge of olfaction in CSFB and their behavioural responses to plant‐derived and other bioactive compounds, as well as to synthetic analogues that attract or repel the pest. Glucosinolates, which are non‐volatile, and their volatile derivatives, isothiocyanates, have been shown to act as phagostimulants and attractants, respectively. Furthermore, companion planting with attractive host plant ‘trap crops’ has potential to suppress and divert pest pressure from the main OSR crop. By contrast, companion planting with non‐host plant species, as well as the application of plant solvent extracts and plant‐derived hormones, has been demonstrated to repel, deter and/or induce antifeedant effects in CSFB, respectively. Although these approaches show great promise, significant knowledge gaps remain regarding the identity of the chemicals involved, dose‐dependent behavioural responses, and the interplay between visual, olfactory and tactile cues in CSFB host selection, feeding and oviposition behaviours. Addressing these gaps could inform the design and development of targeted semiochemical‐based interventions that are effective, environmentally benign and compatible with integrated pest management. © 2026 The Author(s). *Pest Management Science* published by John Wiley & Sons Ltd on behalf of Society of Chemical Industry.

## Graphical abstract

Behavioural manipulation of *Psylliodes chrysocephala* provides promising opportunities for sustainable pest management in oilseed rape systems. This review synthesises evidence on glucosinolates and their volatile isothiocyanate derivatives as phagostimulants and attractants, alongside the repellent or antifeedant effects of non‐host plants, plant extracts and hormone‐related compounds within push–pull strategies.

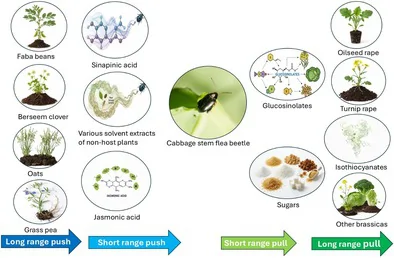

## 1 INTRODUCTION

Oilseed rape (OSR, *Brassica napus* L.) (Brassicaceae), is a globally important crop cultivated primarily for its high‐quality vegetable oil, which is widely used in food production and as a major feedstock for biodiesel. It is the second most important source of vegetable oil worldwide after soybean.^1^, ^2^ However, in recent years, OSR production, and yield, have significantly reduced across Europe, largely attributed to pest management difficulties following the European Union's 2013 restriction on the use of neonicotinoid seed treatment.^3^, ^4^ Although OSR is attacked by a range of invertebrate pests,^5^ the cabbage stem flea beetle (CSFB:, *Psylliodes chrysocephala* L. Coleoptera: Chrysomelidae), is currently ranked as the most economically important pest of winter‐sown OSR.^3^, ^6^

The CSFB is a specialist herbivore of brassicaceous plants.^7^ Adult CSFB undertake seasonal migration flights into newly sown OSR crops in early autumn. They measure 3.2–4.6 mm and are typically black with a blue–green metallic sheen, although a brown form also occurs. They possess enlarged hind femurs that enable their characteristic jumping. Females lay orange, oval eggs in batches, and the resulting larvae develop through three instars while feeding internally within the host plant. The life cycle is presented in Ortega‐Ramos *et al*.^8^ The economic impact of CSFB results from feeding damage caused by both the adult and larval life stages. Adults feed on the cotyledons and leaves of young plants, which threatens crop establishment, and larvae mine stems and petioles, weakening the plant and, in severe cases, causing plant death.^8^, ^9^ Since the neonicotinoid ban, populations of CSFB have increased^10^ and resistance to the only remaining permitted insecticides (pyrethroid sprays) has become widespread.^11^, ^12^ With the lack of effective control, farmers are giving up growing OSR^6^; for example in 2024, UK OSR production was estimated to have declined by approximately 32% falling to around 824 000 t, largely associated with increased pest pressure and consequent yield losses.^13^, ^14^

No fully effective integrated pest management (IPM) strategies for CSFB are currently available; although various approaches are in development (reviewed by Ortega‐Ramos *et al*., 2022 and Hoarau *et al*. 2022^8^, ^15^), they have yet to be optimised and commercially implemented. Cultural control methods like adequate crop rotation and spatial separation^16^, ^17^ and early or late sowing of OSR^18^, ^19^ have been reported to reduce CSFB populations. It has been shown that maintaining a distance of 10 km between previous and current crop fields can reduce beetle pressure on the new crop by up to 76%, indicating that spatial separation could be an effective strategy to suppress infestation^17^ but would be impractical for small farms. Early sowing can help the crop establish before CSFB migration, making it more able to compensate for adult CSFB feeding damage.^20^, ^21^, ^22^ However, predicting CSFB population dynamics remains challenging, making it hard to predict the best time to sow the crop in relation to migration activity^21^; sowing date is therefore not generally recommended as a control strategy.^23^ Biological control options remain limited, with relatively few studies addressing their effectiveness.^15^, ^24^ Although RNA interference (RNAi) options are in development,^25^, ^26^ there are few other insecticidal alternatives on the horizon^27^ and there are also no resistant OSR varieties commercially available that could offer an alternative means of control.^28^, ^29^ This situation clearly requires other complementary strategies to control CSFB.

Push–pull pest management strategies offer considerable potential for sustainable insect control. They represent an IPM approach that manipulates insect behaviour by simultaneously discouraging pests from colonising the main crop (‘push’) and attracting them to alternative plants (‘pull’).^30^ This can be achieved through the use of semiochemicals, (plant‐ or insect‐derived compounds that modify behaviour and development),^30^, ^31^ which can be used via spray or controlled‐release dispenser‐based systems, or expressed directly by plants deployed as strategically designed companion cropping systems, with the aim of disrupting host‐location processes and reducing dependence on synthetic toxicant chemical insecticides.^30^, ^32^ Semiochemicals may be non‐volatile or volatile and operate over short to long ranges, respectively.^33^ Repellent or deterrent semiochemicals could be utilised to ‘push’ CSFB away from OSR crops, while attractive cues simultaneously ‘pull’ them towards baited traps or sacrificial ‘trap crop’ plants, where they are subsequently removed or suppressed, thereby limiting pressure on the main crop.^30^ In this review, we synthesise past and current research on the use of attractants, repellents, deterrents and antifeedants as potential tools for suppressing CSFB in OSR cropping systems and discuss findings related to their olfactory capabilities.

## 2 HOST PLANT LOCATION, SELECTION AND OLFACTION IN CABBAGE STEM FLEA BEETLE

Insects, including CSFB, use a combination of visual and olfactory cues for host plant location, with host plant volatiles playing a central role in their feeding, mating and oviposition behaviours.^34^, ^35^ However, the ability of CSFB to detect and respond to volatile cues from host plants is relatively understudied.^36^, ^37^, ^38^, ^39^ CSFB has well‐defined olfactory sensilla^37^, ^40^ that are highly responsive to the breakdown products of glucosinolates, such as isothiocyanates,^36^, ^37^, ^39^, ^41^ a class of compounds released by cruciferous plants upon tissue damage.^42^, ^43^ The antennal sensilla chaetica of CSFB function primarily through contact, exhibiting both mechanosensory and gustatory roles.^37^ They detect phytochemicals on plant leaf surfaces and respond to mechanical stimulation during antennation, enabling the insect to assess plant palatability without the need to bite. This sensory capability likely explains why CSFB often rejects non‐host plants without chewing them.^7^, ^37^

Although recent findings suggest that CSFB is capable of responding to plant volatile cues,^39^ there is evidence of sexual dimorphism in olfactory responses; only females were found to respond to volatile cues emitted by mechanically damaged seedlings and six‐week‐old OSR plants, whereas undamaged plants elicited no olfactory response.^39^ Although meta‐analyses have shown that male insects are generally less responsive to plant volatiles than females,^44^ no morphological evidence of sexual dimorphism in CSFB antennal sensilla has been found.^40^

Plant volatiles alone are not sufficient to induce oviposition in CSFB, indicating that physical contact with the host plant or short‐range semiochemicals are necessary.^45^ Magnin *et al*.^46^ suggested that physical disruptions and/or volatile cues from non‐host faba bean plants might influence CSFB behaviour, resulting in reduced adult beetle numbers on OSR when grown in combination compared with when OSR was grown with artificial non‐host plants (visually mimicking faba bean with no olfactory cues).^46^ However, following beetle migration and the subsequent atrophy of flight muscles,^47^, ^48^ it is possible that their olfactory sensitivity may weaken, causing older beetles to rely more heavily on visual and gustatory cues, although this hypothesis has not been empirically tested in CSFB. Further research is thus needed to elucidate the specific roles of olfactory and contact cues in mediating the behaviour of both sexes of CSFB during different physiological states.

Further limiting the understanding and application of olfaction in CSFB is the lack of known sex or aggregation pheromones. The secretion of sex pheromones by reproductively active males has been suggested.^49^ The low volatility of the compounds, together with behavioural observations of male antennal movements during mating, led to the suggestion that the compounds may not act as an attractant but as an arrestant aphrodisiac, and that proximal contact may be a prerequisite for mating. However, this has yet to be fully corroborated, and the compounds have not been identified. For this reason, plant‐based push–pull strategies currently offer the greatest potential, at least in the short term, as outlined in the following sections.

## 3 PUSH ‐ REPELLENTS, ANTIFEEDANTS AND DETERRENTS

In push–pull management systems, the ‘push’ element is designed to minimise pest infestation by introducing stimuli that discourage pests from approaching, settling on and/or feeding on the main crop. This can be achieved using repellent cues that disrupt host location, as well as antifeedant/deterrent compounds that suppress feeding or oviposition upon contact. These can be plant secondary metabolites and even plant hormones that interfere with behavioural and sensory processes, thereby reducing or diverting pest pressure away from the main crop.^30^, ^33^ To this end, plant‐based semiochemicals represent a promising strategy for the management of CSFB while providing environmentally sustainable alternatives to synthetic insecticides push.^30^, ^31^ Table 1 summarises repellent, deterrent and antifeedant compounds that may play a role in suppressing CSFB in OSR cropping systems.

**Table 1:** Repellents, deterrents and antifeedants for cabbage stem flea beetle (CSFB; *Psylliodes chrysocephala*) with potential for use in oilseed rape (OSR) crop protection

Source	Effect	References
Oats, wheat, Berseem clover (*Trifolium alexandrinum*), faba bean (*Vicia faba*), grass pea (*Lathyrus sativus*)	In field studies non‐host companion plants significantly suppressed populations in OSR crops, but mechanism and compounds involved unknown	46, 52, 53, 54
Non‐host plants: *Helianthus annuus*, *Vicia faba*, *Pisum sativum*, *Plantago major*, *Crambe maritima*	In laboratory studies, non‐host plants were rejected even when they contained glucosinolates or when glucosinolates were artificially applied to glucosinolate‐free plants. Antifeedant compounds suggested to include polyacetylenes and sesquiterpene lactones for *H*. *annuus* and non‐protein amino acids for *V*. *faba* and *P*. *sativum*	7, 38
White mustard (*Sinapis alba*), Kohlrabi (*Brassica oleracea* var. gongylodes). Cauliflower (*Brassica oleracea* var. botrytis), radish (*Raphanus sativus* L), turnip rape (*Brassica rapa* subsp. *oleifera* DC)	Companion planting OSR with white mustard significantly reduced adult population	52, 53 7
	White mustard, Kohlrabi cauliflower, radish and turnip rape had lower preference indices in laboratory feeding tests than oilseed rape	
Solvent extracts of *Iberis amara*, *Hesperis matronalis*, *Capsella bursa‐pastoralis*, *Erysimum cheiri*, *Hesperis matronalis*, *Lunaria annua*, *Erysimum linifolium*, *Plantago major* and *Crambe maritima* applied on oilseed rape plants	In laboratory studies, at least one extract of each plant significantly reduced feeding	7
	*Erysimum* spp. contain cardiac glycosides, *L*. *annua* contains saponins and alkaloids, and *I*. *amara* contains cucurbitacins; all compounds known to inhibit feeding by Chrysomelidae	
Azadirachtin	Reduced feeding by adults in a laboratory study	60
Jasmonic acid (JA)‐treated oilseed rape	Reduced feeding on plants treated with low concentrations of JA, compared with untreated plants in laboratory studies. Jasmonate is known to induce phenolic compounds including phenylpropanoids	67
Sinapinic acid (a phenyloropanoid phenolic)	Present in high concentrations in *Sinapis alba* which suffered significantly less feeding damage than oilseed rape. Acted as a feeding deterrent when applied to OSR leaf discs in laboratory studies	73

### 3.1 Long range

#### 3.1.1 Companion planting as push

Companion planting involves growing additional plant species alongside or within the crop to indirectly suppress herbivore pest pressure by repelling them, disrupting host‐location cues or masking OSR attractiveness through chemical or physical interference.^50^, ^51^ For example, OSR undersown with companion plants, such as clover or cereals (oats or wheat), destroyed after the immigration phase of the beetle, significantly reduced both larval and adult CSFB populations.^52^ However, variations across years were observed, with some seasons having no significant reduction in crop damage by CSFB due to poor companion seedling establishment in prolonged drought conditions, that also created competition for water and affected the main crop.

Leguminous plants, including berseem clover (*Trifolium alexandrinum*), planted with OSR significantly reduced CSFB abundance and damage, with effectiveness increasing over time as the clover advanced in growth stage/biomass.^52^, ^53^, ^54^ Furthermore, intercropping OSR with faba beans (*Vicia faba*) and pea grass (*Lathyrus sativus*) resulted in significantly fewer CSFB larvae than in control plots.^54^ This approach also led to a reduction in the population of the pollen beetle (*Brassicogethes aeneus*) and stem weevil (*Ceutorhynchus napi*), two other major pests of OSR. Magnin *et al*.^46^ compared CSFB responses in OSR plots containing artificial faba bean plants, real faba bean intercrops or OSR monocrops. No reduction in adult beetle populations in plots with artificial plants was found, whereas a significant decrease was recorded in plots containing real faba beans, suggesting that olfactory disruptions from the faba bean non‐host plants was an important mechanism of the success of companion planting to reduce pest populations. Whether the mechanism was volatile masking or actual repellency, as well as the role of visual cues remains unclear and needs further validation. Interestingly, increased soil nitrogen levels are associated with reduced damage from CSFB, *B*. *aeneus* and *Ceutorhyncus* spp.^18^ Given that leguminous plants naturally enrich the soil with nitrogen, it is plausible that elevated nitrogen uptake in OSR altered plant chemistry and thus reduced feeding or oviposition, although this has not been directly demonstrated.

Another advantage of companion planting is that the additional species increase plant biodiversity and associated habitats, which have biodiversity benefits.^55^, ^56^ Furthermore, the companion plants may release semiochemicals or possess other traits that positively influence the host‐finding behaviour of natural enemies further facilitating biological control.^57^, ^58^

### 3.2 Short range

#### 3.2.1 Plant extracts as antifeedants and deterrents

Extracts of plants have long been used to repel, deter or kill insects on crop plants with extracts from the Neem tree (*Azadirachta indica*), and azadirachtin – the active component – being well‐known antifeedants for insects.^59^ However, although feeding trials with a commercially available product reported reduced feeding by CSFB compared with untreated controls, variations were not statistically different and were inconsistent with dose.^60^ When the non‐host plants *Helianthus annuus* (Asteraceae), *Vicia faba* and *Pisum sativum* (Fabaceae) were presented to CSFB, starved beetles rejected the plants and did not feed, even when the glucosinolate sinigrin, a feeding stumulant was applied.^7^ Feeding inhibitors in the solvent extracts were suggested to include polyacetylenes and sesquiterpene lactones for *H*. *annuus* and non‐protein amino acids for *V*. *faba* and *P*. *sativum*, compounds with toxic and antifeedant properties.^7^ Furthermore, solvent extracts from the brassicaceous species *Iberis amara*, *Hesperis matronalis*, *Capsella bursa‐pastoralis*, *Erysimum cheiri*, *Lunaria annua*, *Erysimum linifolium* and *Crambe maritima*, as well as from the non‐brassica *Plantago major*, inhibited the feeding activity of adult CSFB when applied to OSR plants.^7^ *Erysimum* species contain cardiac glycosides, compounds known to inhibit feeding in Chrysomelidae^61^, ^62^; *L*. *annua* contains saponins and alkaloids, both of which are antifeedants for other flea beetle species, such as *Phyllotreta striolata*,^61^, ^62^, ^63^ whereas *I*. *amara* contains cucurbitacins that inhibit feeding by *Phyllotreta nemorum*.^61^, ^62^, ^63^ Although the authors did not specifically relate these compounds to the deterrent effect, cucurbitacins may be responsible for the feeding reduction observed.^7^, ^64^ More research is needed to investigate the compounds responsible for these observations and the duration of their effects. Plant extracts are readily accessible, cost‐effective and stable, showing potential for development in pest management; however, these properties need to be evaluated thoroughly before practical application.

#### 3.2.2 Plant hormones and hormone‐modulating compounds as repellents and antifeedants

Feeding by CSFB on OSR was significantly reduced when plants were treated with jasmonic acid (JA), a plant hormone usually activated in response to herbivory,^65^, ^66^ compared with untreated plants or those previously fed on by conspecifics.^67^ This effect was stronger at the cotyledon leaf stage than at the true leaf stage.^67^ The reduction in feeding was attributed to JA‐induced alterations in the plant's glucosinolate profile, resulting in a 6.4‐fold increase in glucosinolate concentrations, levels that may exceed the beetles’ tolerance threshold.^67^, ^68^, ^69^, ^70^ In addition to deterring CSFB, JA‐induced glucosinolate concentrations may also enhance resistance to generalist herbivores, such as slugs,^67^ and reduce plant susceptibility to fungal pathogens.^71^ Treatment with jasmonate increases the biosynthesis of phenolic compounds in plants, indicating that these phytohormones act as effective elicitors of phenylpropanoid derived secondary metabolites.^72^ Some of the phenolic compounds induced may be antifeedant.^66^

Sinapinic acid (also known as sinapic acid), a phenylpropanoid phenolic naturally occurring in the Brassicaceae, acted as a feeding deterrent to CSFB.^73^ It was present in high concentrations in *Sinapis alba* seedlings, which exhibited reduced feeding compared with OSR seedlings, and the deterrent effect was further confirmed when sinapinic acid was applied to OSR leaf discs, resulting in a significant reduction in beetle feeding.^73^ Furthermore, related hydroxycinnamic acids have been shown to play a key role in the modulation or amplification of plant hormone signalling and contribute to disease resistance by reinforcing plant cell walls.^74^ Interestingly, OSR seedlings grown from old seeds (stored for more than 5 years) exhibited significantly reduced CSFB feeding damage compared to OSR seedlings from freshly harvested seed. Although metabolomic profiling revealed shifts (e.g. an increase in erucic acid and a decrease in fumaric acid) in comparison with fresh seeds, the reason(s) for the antifeedant effect remain unidentified. If these can be characterised, they, like sinapinic acid, may represent novel targets for breeding programmes or exogenously applied deterrents within a push–pull framework.

## 4 PULL ‐ ATTRACTANTS AND STIMULANTS

In OSR cropping systems, the ‘pull’ components in a push–pull pest management strategy are intended to draw pests away from infesting the main crop by exploiting a strong preference for particular host plants and/or attractive chemical cues.^30^ This is typically achieved through the deployment of baited traps or trap crops that provide stronger attractant cues, phagostimulatory or oviposition cues than the main crop itself.^30^ By drawing adult CSFB away from establishing OSR and concentrating their populations in defined areas, pull strategies can reduce early crop damage via reduced feeding and can also facilitate more targeted pest control.^30^, ^75^, ^76^ In addition to reducing reliance on synthetic insecticides, trap cropping can contribute ecological benefits, including support for natural enemies and enhanced habitat complexity, making it a potentially sustainable technique for pest management in OSR systems.^30^, ^76^, ^77^ However, although trap cropping offers promising advantages, it often results in a reduced cropping area and therefore reduced overall yield for a given field, and increased management complexity.

CSFB feeds mainly on plants in the Brassicaceae family,^7^, ^67^ despite the presence of glucosinolates which are defence compounds toxic to generalist feeders.^78^ Upon plant tissue damage, these compounds are hydrolysed into toxic isothiocyanates (ITCs), which typically repel generalist herbivores;^78^ however, CSFB are attracted to ITCs.^39^, ^79^ CSFB is capable of sequestering glucosinolates throughout its life cycle and also possess mechanisms to detoxify them and ITCs during feeding.^69^, ^70^ Glucosinolates typically function as short‐range cues because of their non‐volatile nature, primarily by stimulating feeding, whereas ITCs are volatile and can act as long‐range attractants.^38^, ^79^, ^80^ In this section, we examine long‐ and short‐range semiochemicals that attract CSFB or function as phagostimulants, respectively, and consider the potential application of plant‐derived compounds and their synthetic analogues in developing sustainable pest management strategies. A summary of these compounds is provided in Table 2.

**Table 2:** Attractants/phagostimulants for cabbage stem flea beetle (CSFB; *Psylliodes chrysocephala*) with potential for use in oilseed rape (OSR) crop protection

Source	Effect	References
Chinese cabbage (*Brassica rapa pekinensis*), garden rocket (*Eruca vesicaria* L.), watercress (*Nasturtium officinale* R. Br.)	In a study comparing multiple *Brassica* species, *Nasturtium officinale* had the highest mean adult feeding preference, followed by Chinese cabbage and garden rocket. In another laboratory study, Chinese cabbage had greater acceptability than OSR or *Iberis amara*	7, 45
		45
Turnip rape (*Brassica rapa*)	Attractive for adult feeding and oviposition. Potential use as trap crop	7, 51, 52, 84, 91
	Larvae were supported better on turnip rape than OSR indicating phagostimulants or nutritive differences	97
White mustard (*Sinapis alba*)	Larval weight was less than in OSR and development was slower. Poor plant survival overwinter shows potential as a ‘dead‐end’ trap crop	97
Excised or mechanically damaged oilseed rape seedlings	Adults attracted to baited yellow water traps in the field (response comparable to baits with synthetic allyl isothiocyanates) in field trapping and in olfactometer bioassays in the laboratory	38, 79
	Strongly attractive to female adults in laboratory olfactometer bioassays	39
Methanol extract of oilseed rape	Electrophysiological recordings of the sensilla chaetica revealed it was the most effective stimulus compared to different glucosinolates	37
Sugars: glucose and fructose	Sugars acted as a phagostimulant	37, 38, 80
Carbohydrates	Increased levels of digestible carbohydrates in oilseed rape than Chinese cabbage were related to increased oviposition rates	45
Glucosinolates, including butenyl and particularly indole glucosinolates including glucobrassicin and sinalbin. progoitrin and 4‐hydroxyglucobrassicin.	Plants that contained glucosinolates, including young oilseed rape, were preferentially fed upon by both adults and larvae indicating phagostimulatory effects	7, 37, 38, 39, 80, 81, 89, 90
	Dose‐dependent effects of sinalbin in laboratory feeding tests on adults	73
	Concentrations of progoitrin and 4‐hydroxyglucobrassicin were positively correlated with larval weight	97
Glucosinolate‐derived volatile organic compounds including isothiocyanates (ITCs): 3‐butenyl and 4‐pentenyl ITC, allyl ITC, 2‐penylethyl ITC, indole‐3‐carbinol, sec‐butyl ITC.	Traps in the field caught significantly more adults when baited with isothiocyanates	36, 39, 81
A mixture of 3‐butenyl and 4‐pentenyl and 2‐phenylethyl ITC	Electroantennography studies indicate a high proportion of adult antennal sensilla responded strongly to isothiocyanates	36
	Female adults responded strongly to 2‐phenylethyl ITC in olfactometer studies in the laboratory	39
indole‐3‐carbinol, allyl ITC, sec‐butyl ITC, and 2‐phenylethyl ITC	Synthetic blend simulating mechanically damaged oilseed rape seedlings was attractive to female adults in olfactometer bioassays in the laboratory	39

### 4.1 Long‐range attractants

Field studies have demonstrated that CSFB are attracted to selected host plant volatiles. In experiments using yellow water traps, significantly more migrating adults were captured in traps baited with synthetic 4‐pentenyl, 3‐butenyl and 2‐phenylethyl ITC, as well as to excised OSR seedlings.^38^, ^79^ Traps containing allyl ITC were also highly attractive to CSFB^37^, ^80^, ^81^ and allyl ITC is a particularly promising attractant candidate for long‐range lures for CSFB owing to its low cost and wide availability – given its broad industrial use, including in the food industry.^82^ Similarly, attraction in an olfactometer to the odours of 30 mechanically damaged OSR seedlings was reported^39^ (undamaged seedlings elicited no significant response). This is consistent with Bartlet *et al*.^79^ who observed that adult CSFB responded only when exposed to baits containing 50 OSR seedlings, suggesting that under field conditions, a substantial accumulation of volatile organic compounds from OSR plant material may be required to initiate attraction.

Several studies have demonstrated the benefits of using trap crops for pest control and the practice is gaining increasing attention among OSR farmers as a promising strategy to manage CSFB.^83^ Turnip rape (*Brassica rapa*) used as a border strip was highly effective in reducing CSFB larval pressure in OSR compared with plots without trap crops, indicating clear diversion of oviposition away from the main crop.^84^ Larval abundance was also higher in the turnip rape borders than in OSR plants in the borders of monocrops, indicating a preference for turnip rape.^84^ This work was supported by Coston *et al*.^51^ who also demonstrated reduced adult feeding in OSR plots protected by turnip rape. However, the mechanism of action remains poorly understood, and further investigation could help optimise its use. Contrary to these studies, no significant reduction in CSFB pressure was reported in another study when turnip rape was used as a trap crop, although this outcome was attributed to the small plot size used and exceptionally high beetle populations during the study period, which overwhelmed both plant species.^52^ Therefore, it is important to note that during periods of high pest pressure, trap crops alone may provide limited protection unless complemented by additional push strategies, such as incorporating non‐host plants alongside host crops (discussed in Section 3.1.1).

Despite the potential benefits of trap cropping in OSR, the fact that the trap crop species typically belong to the same Brassicaceae family can create management complications. When established as undersown mixtures or intercrops, they may be difficult to control or remove once the main crop is established, necessitating selective herbicides that target the trap crop without harming the OSR main crop.^51^ Moreover, the precise olfactory, gustatory or visual cues driving CSFB responses to the trap crops remain unclear. A better understanding of these mechanisms could support the development of robust, targeted semiochemical‐based strategies to improve the protection of OSR.

It has been proposed^85^ that the most effective strategy for deploying semiochemicals is to target sites where the pests overwinter or aestivate by placing baited traps emitting attractive volatiles near these locations. Emerging CSFB adults could then be lured and captured at close range before they migrate into newly sown OSR crops, significantly reducing incoming populations, especially given that CSFB aestivate at field margins, under leaf debris or in wooded areas away from OSR.^86^ It is also important to note that while certain synthetic compounds, such as ITCs, have shown potential as attractants, their use raises concerns, since some may be toxic or even carcinogenic to humans.^87^, ^88^ Consequently, their deployment would require carefully designed formulations to reduce the risk of unintended impacts on users and non‐target organisms.

### 4.2 Short‐range attractants and phagostimulants

#### 4.2.1 Glucosinolates as phagostimulants

Short‐range attractants are chemical cues that influence insect behaviour at close distances, usually after an insect has landed on or contacted a potential host. These cues are typically non‐volatile or only weakly volatile and function primarily as phagostimulants or oviposition stimulants, or elicit host acceptance, rather than mediating long‐distance orientation.^33^ Both larval and adult CSFB feed only on plants containing glucosinolates.^7^, ^38^ Feeding activity in adult CSFB was related to the amount of total glucosinolates^89^, ^90^ but studies have indicated that some classes, like butenyl and indole glucosinolates – the latter being induced by mechanical damage and by CSFB feeding – are more important than others.^89^, ^90^ Work by Bartlet *et al*. showed that glucosinolates (and sugars) act as phagostimulants, because they induced feeding of CSFB when presented in an agar‐based artificial diet.^38^, ^80^ However, when non‐host plants were treated with sinigrin (allyl glucosinolate), the beetles exhibited no feeding activity.^7^ This suggests that feeding behaviour is not solely driven by glucosinolates alone but instead relies on multiple sensory inputs and additional host‐specific traits such as surface wax chemistry and other secondary metabolites inherent in Brassicaceae.^7^, ^80^, ^91^

The antennal sensilla chaetica of CSFB have been shown to elicit a stronger response to the glucosinolate glucobrassicin compared with epiprogoitrin, glucobrassicanapin and sinigrin.^37^ This was also supported by a feeding assay, which identified glucobrassicin as a more effective feeding stimulant, followed by sinigrin.^80^ Another study investigated eight different OSR lines to determine whether variation in glucosinolate content influenced CSFB feeding.^92^ The findings revealed no relationship between glucosinolate content and feeding activity in the different plant accessions, suggesting that glucosinolates may act primarily as feeding stimulants rather than true short‐range attractants.

Synergistic and antagonistic effects, as well as dose‐dependence, should be taken into account. As shown by Rüde *et al*.,^73^ a metabolite mixture applied to leaf discs had a deterrent effect on CSFB feeding. This deterrent effect was neutralised by the addition of a small amount of the glucosinolate sinalbin, whereas a large amount of sinalbin, as found in *S*. *alba* cotyledons, left the deterrent effect unaffected. A dose‐dependent effect of sinalbin was also reported by Bodnaryk^93^ for *Phyllotreta cruciferae*. Growth stage‐dependent effects should also be considered when selecting candidate pull plant species, as illustrated by *S*. *alba*; although *S*. *alba* may be attractive to CSFB at later growth stages, it exhibited comparably high resistance to CSFB feeding at the seedling stage (BBCH 10) in no‐choice laboratory and semi‐field experiments.^73^

#### 4.2.2 Host plant feeding preference

Experimental studies conducted under controlled, limited‐choice conditions have shown that starved beetles may feed on non‐host plants such as nasturtium (*Tropaeolum majus*) (Tropaeolaceae) and white mignonette (*Reseda alba*) (Resedaceae), both of which contain glucosinolates. Minimal feeding was also observed on non‐host plants that contain glucosinolates, such as wild candytuft (*Iberis amara*), dame's rocket (*Hesperis matronalis*), shepherd's purse (*Capsella bursa‐pastoris*), English wallflower (*Cheiranthus cheiri*), spiny spiderflower (*Cleome spinosa*), honesty (*Lunaria annua*) and slender leaved wallflower (*Erysimum linifolium*).^7^ It is important to note that this feeding on non‐host plants may have been influenced by several experimental factors; for example, the test plant material was presented as leaf discs rather than intact whole plants. Damaged plant tissues can have altered biochemistry due to induction of defence mechanisms^78^, ^94^ and altered physical texture, potentially making it more acceptable to the beetles than under natural conditions.^7^

In a study assessing the feeding preference of adult CSFB across different *Brassica* species, watercress (*Nasturtium officinale*) elicited the highest mean adult preference at 67.4, followed by Chinese cabbage (*Brassica rapa pekinensis*) (62.9) and garden rocket (*Eruca vesicaria*) (62.2).^7^ Turnip (*Brassica rapa* subsp. *rapa*), pak choi (*Brassica rapa* subsp. *chinensis*), black mustard (*Brassica nigra*) and brussels sprouts (*Brassica oleracea* var. *gemmifera* DC) had preference mean values of 55.1, 52.2, 51.7 and 51.7 respectively, exhibiting similar levels of acceptability with OSR. Furthermore, kohlrabi (*Brassica oleracea* var. *gongylodes*) and white mustard (*Sinapis alba*) had a mean adult feeding preference of 49.4 and 45.6, respectively. In comparison, cauliflower (*Brassica oleracea* var. *botrytis*) (35.8), turnip rape (*Brassica rapa* subsp. *oleifera* DC) (37.1) and radish (*Raphanus sativus*) (37.6) showed somewhat lower levels of preference. The mechanisms for these preferences were not identified but are likely driven by plant chemistry. Although *Nasturtium officinale* was strongly preferred as a whole plant, it has no practical application in trap cropping because it is an aquatic plant species. *Nasturtium officinale* has a high glucosinolate content but is also rich in polyphenols.^95^ What is of interest are the volatile compounds (e.g. myristicin, α‐terpinolene, limonene),^95^ which have not been evaluated as attractants to CSFB. It was recently demonstrated that Chinese cabbage, which is known to be rich in glucosinolates (e.g. progoitrin, gluconapin), polyphenolics (e.g. sinapic acid), carotenoids and tocopherols,^96^ was more acceptable to CSFB for feeding than OSR; however, the oviposition rate was significantly higher after feeding on OSR than Chinese cabbage, indicating that dietary requirements might play an important role in egg‐laying.^45^ OSR was found to have higher concentrations of soluble carbohydrates than Chinese cabbage and these may provide specific nutrients and potentially also physiological cues upon contact essential for initiating oviposition.^45^

Larval host preference in CSFB, measured as the number of larvae penetrating host plants when presented with the plants, was studied.^7^ Garden rocket (*Eruca vesicaria*) was the most preferred host, with all larvae entering the plant to begin feeding. This was followed by Chinese cabbage, kohlrabi, wild mustard (*Sinapis arvensis*), pak choi, Pe‐tsai or Nepa cabbage (*Brassica rapa* subsp. *pekinensis*) and stock (*Matthiola incana*). Slightly lower levels of larval penetration were observed in broccoli (*Brassica oleracea* var. *italica*), turnip and radish (*Raphanus sativus*), followed by OSR, cauliflower, black mustard, white mustard and garlic mustard (*Alliaria petiolata*). However, under field conditions, host selection is primarily driven by adult behaviour, with oviposition typically occurring near previously infested plants, thereby limiting larval host choices. Consequently, it is unclear whether these plant species would effectively suppress larval populations under field conditions. However, in another study, Döring and Ulber^97^ found that turnip rape generally supported better larval development compared with OSR and white mustard. This was evidenced by significantly higher larval weights on turnip rape, indicating its superior host quality for larval development compared to the other brassicaceous species. If planted as a trap crop, turnip rape plants should therefore be destroyed before larval development is completed because this species could act as a source of the pest. White mustard is an interesting candidate pull species for push–pull systems, as it is inexpensive and easy to cultivate. Furthermore, under typical Central European conditions it will not overwinter before setting seed, effectively making it a 'dead end' trap crop for CSFB larvae (which begin to exit the plant to pupate from February).^5^ In addition, larval weight was lower and larval mortality was higher than on *B*. *napus*.^97^

## 5 CONCLUSION

Semiochemicals, particularly plant‐derived compounds and their synthetic analogues, offer considerable potential for IPM in OSR by modifying the behaviour of CSFB through long‐range cues acting as repellents (‘push’) and attractants (‘pull’), while short‐range compounds with antifeedant (‘push’) or phagostimulatory (‘pull’) effects operate at the scale of host acceptance and feeding. Deterrent, repellent and antifeedant push stimuli such as plant extracts, JA and the use of non‐host companion plants, have been shown to significantly reduce or suppress adult feeding. Attractant stimuli, including indole glucosinolates, trap crops and traps baited with ITCs, function by exploiting strong host preference and phagostimulatory cues that divert pest feeding and oviposition away from the main crop by providing a more attractive alternative, thereby condensing pest populations in controlled areas where they are afterwards managed or removed. The use of push–pull systems may offer advantages over sole reliance on chemical insecticides by reducing synthetic pesticide usage and crop damage during the vulnerable establishment phase. However, land‐use trade‐offs associated with companion or trap cropping, the cost of establishing effective push–pull systems and variability in pest pressure remain insufficiently quantified or studied for CSFB. Importantly, behavioural manipulation‐based strategies are less likely to impose strong selection pressure for resistance compared with synthetic insecticides. However, repeated activation of plant defence pathways such as the use of JA to reduce attraction in OSR may affect plant growth and eventually affect yield. Overall, although no fully optimised push–pull system for CSFB is currently available, the evidence synthesised in this review indicates strong potential for strategies based on pest behaviour manipulation which may act synergistically, improving the overall effect in comparison to use of either push or pull elemens alone.

Several critical knowledge gaps exist. The physiological and behavioural responses of CSFB, including sex‐specific olfactory sensitivity, the relative contributions of olfactory, visual and tactile cues, and dose‐dependent effects of semiochemicals, are not fully understood. To our knowledge, no studies have rigorously assessed the use of essential oils against the pest, and sex or aggregation pheromones have not yet been identified and chemically characterised. This review also highlights the need for further research to identify the mechanisms responsible for the attraction, repulsion and deterrent effects reported in previous studies. Future research should prioritise studies resolving dose‐dependent responses to key semiochemicals and conducting robust multi‐year field evaluations to assess the persistence, durability and practical effectiveness of semiochemicals as well as their synergy and/or compatibility with existing agronomic practices in OSR systems. With increased research effort, push–pull strategies have the potential to become a powerful tool for sustainable CSFB management and reducing dependence on synthetic insecticides.

## CONFLICT OF INTEREST

The authors declare no conflicts of interest.

## Data Availability

Data sharing not applicable to this article as no datasets were generated or analysed during the current study.
